# Cl^−^ homeodynamics in gap junction-coupled astrocytic networks on activation of GABAergic synapses

**DOI:** 10.1113/jphysiol.2013.257162

**Published:** 2013-06-03

**Authors:** Kiyoshi Egawa, Junko Yamada, Tomonori Furukawa, Yuchio Yanagawa, Atsuo Fukuda

**Affiliations:** 1 Department of Neurophysiology, Hamamatsu University School of Medicine Hamamatsu 431-3192, Japan; 2 Department of Neurophysiology, Hirosaki University Graduate School of Medicine Hirosaki 06-8560, Japan; 3 Department of Genetic and Behavioral Neuroscience, Gunma University Graduate School of Medicine Gunma 371-8510, Japan

## Abstract

The electrophysiological properties and functional role of GABAergic signal transmission from neurons to the gap junction-coupled astrocytic network are still unclear. GABA-induced astrocytic Cl^−^ flux has been hypothesized to affect the driving force for GABAergic transmission by modulating [Cl^−^]_o_. Thus, revealing the properties of GABA-mediated astrocytic responses will deepen our understanding of GABAergic signal transmission. Here, we analysed the Cl^−^ dynamics of neurons and astrocytes in CA1 hippocampal GABAergic tripartite synapses, using Cl^−^ imaging during GABA application, and whole cell recordings from interneuron–astrocyte pairs in the stratum lacunosum-moleculare. Astrocytic [Cl^−^]_i_ was adjusted to physiological conditions (40 mm). Although GABA application evoked bidirectional Cl^−^ flux via GABA_A_ receptors and mouse GABA transporter 4 (mGAT4) in CA1 astrocytes, a train of interneuron firing induced only GABA_A_ receptor-mediated inward currents in an adjacent astrocyte. A GAT1 inhibitor increased the interneuron firing-induced currents and induced bicuculline-insensitive, mGAT4 inhibitor-sensitive currents, suggesting that synaptic spillover of GABA predominantly induced the astrocytic Cl^−^ efflux because GABA_A_ receptors are localized near the synaptic clefts. This GABA-induced Cl^−^ efflux was accompanied by Cl^−^ siphoning via the gap junctions of the astrocytic network because gap junction inhibitors significantly reduced the interneuron firing-induced currents. Thus, Cl^−^ efflux from astrocytes is homeostatically maintained within astrocytic networks. A gap junction inhibitor enhanced the activity-dependent depolarizing shifts of reversal potential of neuronal IPSCs evoked by repetitive stimulation to GABAergic synapses. These results suggest that Cl^−^ conductance within the astrocytic network may contribute to maintaining GABAergic synaptic transmission by regulating [Cl^−^]_o_.

**Key points**

- Astrocytes encapsulate GABAergic synapses and express GABA_A_ receptors and GABA transporters. They are tightly coupled by gap junctions, and are referred to as the gap junction-coupled astrocytic network.
- With higher [Cl^−^]_i,_ GABA application can mediate bidirectional Cl^−^ fluxes in astrocytes, Cl^−^ efflux via GABA_A_ receptors, and Cl^−^ influx along with GABA uptake via GABA transporters.
- We focused on the Cl^−^ dynamics of the astrocytic network under GABAergic synapse transmission. Spillover of GABA predominantly induced Cl^−^ efflux via GABA_A_ receptors, presumably because they are localized more closely to the synaptic cleft.
- GABA_A_ receptor-mediated currents were propagated via gap junctions within the astrocytic network. These results indicate that Cl^−^ efflux from astrocytes mediated by GABAergic transmission is homeostatically maintained within gap junction-coupled astrocytic networks.
- Blockage of gap junctional coupling by octanol promoted the collapse of the driving force for neuronal inhibitory transmission during intense activation of GABAergic synapses. Thus, the astrocytic network may play a role in maintaining GABAergic transmission by regulating [Cl^−^]_o_.

## Introduction

Astrocytic processes encapsulate synapses tightly and express receptors (Verkhratsky & Steinhauser, 2000) and transporters (Eulenburg & Gomeza, 2010) for a variety of neurotransmitters. This enables astrocytes to participate in information processing of the central nervous system and to modulate neuronal signal transmission. The expression of GABA_A_ receptors in astrocytes has been demonstrated in cell culture (Kettenmann *et al.* 1984*b*; Backus *et al.* 1988) and *in situ* in various brain regions (MacVicar *et al.* 1989; Muller *et al.* 1994). In contrast to neurons, their activation causes Cl^−^ efflux, which results in astrocytic membrane depolarization, in cell culture (Kettenmann *et al.* 1987; Backus *et al.* 1988) and *in situ* (MacVicar *et al.* 1989; Bekar & Walz, 2002) throughout postnatal development. This depolarization stems from the high [Cl^−^]_i_ maintained by the activity of the Na^+^/K^+^/2Cl^−^ cotransporter (NKCC1) (Yan *et al.* 2001), but the physiological significance of astrocytic GABA_A_ receptor activation remains to be elucidated. GABA_A_ receptor-mediated depolarization induces morphological changes (Matsutani & Yamamoto, 1997) and a rise in cytosolic [Ca^2+^]_i_ (Bernstein *et al.* 1996; Meier *et al.* 2008), implying a regulatory role in the physiological functions of astrocytes.

Kettenmann *et al.* (1987) hypothesized that Cl^−^ efflux from astrocytes could buffer the [Cl^−^]_o_ of the encapsulating synapse and maintain GABAergic neuronal transmission. This hypothesis has been afforded greater importance by cumulative evidence illustrating the dynamics of the driving force for neuronal GABAergic transmission during intense GABA_A_ receptor activation (Staley *et al.* 1995; Kaila *et al.* 1997; Staley & Proctor, 1999). Synaptically activated Cl^−^ accumulation via GABA_A_ receptors causes collapse of the neuronal [Cl^−^]_o_/[Cl^−^]_i_ gradient, inducing transient GABA-mediated depolarization (Isomura *et al.* 2003). This depolarization might be moderated by Cl^−^ efflux via astrocytic GABA_A_ receptors activated by spillover of GABA.

To estimate astrocytic participation in synaptic Cl^−^ homeodynamics, the interactions among presynaptic GABAergic neurons, postsynaptic neurons and encapsulating astrocytes should be revealed. Astrocytic GABA_A_ receptors may act as a siphon that counterbalance the [Cl^−^]_o_ regulation of postsynaptic GABA_A_ receptors and presynaptic and astrocytic GABA transporters (GATs), the latter co-transporting Cl^−^ along with GABA (Kanner & Schuldiner, 1987). In addition, gap junctional coupling that equalizes the ion concentration within the astrocytic network (Rose & Ransom, 1997) may contribute to the buffering of [Cl^−^]_o_.

The properties of GABAergic neuron-to-astrocyte signal processing are still unclear because few studies have investigated the astrocytic responses induced by presynaptic GABAergic activation. Electrical stimulation of presynaptic fibres evokes concomitant K^+^ currents in astrocytes (Bergles & Jahr, 1997; Kinney & Spain, 2002), which hinder the precise evaluation of kinetically slow astrocytic GABAergic responses. To overcome this, we directly evaluated single GABAergic neuron–astrocyte signal transmission in the mature CA1 hippocampus by performing dual whole cell patch clamp recordings on each component. In comparison with the results of GABA application, we illustrate that GABA spillover activates astrocytic GABA_A_ receptors localized near the synaptic clefts, and that their signals propagate to neighbouring astrocytes via gap junctions. Furthermore, we show data suggesting that such homeostatic dynamics of Cl^−^ within the astrocytic network might contribute to maintain intense neuronal GABAergic transmission by regulating [Cl^−^]_o_.

## Methods

### Ethical approval

All experiments conformed to the guidelines issued by Hamamatsu University School of Medicine on the ethical use of animals for experimentation.

### Slice preparation

Experiments were carried out on acute hippocampal slices prepared from 40 male, 19–30-day-old C57BL/6 heterozygous *GAD67*-green fluorescent protein (GFP) knock-in (GAD67^+/GFP^) mice (Tamamaki *et al.* 2003). Animals were killed by decapitation under deep anaesthesia using halothane, and hippocampal slices (350 μm thick) were cut on a microslicer (VT-1000S; Leica Microsystems, Wetzlar, Germany) in ice-cold modified artificial cerebrospinal fluid (ACSF) containing (in mm): 220 sucrose, 2.5 KCl, 1.25 NaH_2_PO_4_, 12.0 Mg_2_SO_4_, 0.5 CaCl_2_, 26.0 NaHCO_3_ and 30.0 glucose, pH 7.4 when gassed with 95% O_2_/5% CO_2_. Following sectioning, the slices were kept at room temperature for >1 h before they were used for experiments in standard ACSF solution consisting of (in mm) 126 NaCl, 2.5 KCl, 1.25 NaH_2_PO_4_, 2.0 MgSO_4_, 2.0 CaCl_2_, 26.0 NaHCO_3_ and 20.0 glucose, pH 7.4 when gassed with 95% O_2_/5% CO_2_. For patch clamp recording experiments in astrocytes, slices were incubated in ACSF that contained 100 nm sulforhodamine 101 (SR101) for 30–40 min at room temperature to identify astrocytes (Nimmerjahn *et al.* 2004).

### Electrophysiology

A slice was placed on the base of a recording chamber located on the stage of a microscope (ECLIPSE, Nikon Tokyo, Japan, for experiments with photo-uncaging systems, or BX61, Olympus Tokyo, Japan, for other experiments) and continuously perfused with oxygenated ACSF at a flow rate of 2 ml min^−1^ at 30°C. The GABA_B_ receptor blocker, CGP55845 (3 μm), was routinely applied in the extracellular solution. The cell images were viewed on a monitor via a 40× water immersion objective lens with an infrared differential interference contrast filter and a charge coupled device (CCD) camera (ORCA-ER C4742-95 or EMCCD #C9100-02; Hamamatsu Photonics, Hamamatsu, Japan). Patch pipettes were fabricated from borosilicate capillary tubing of 1.5 mm diameter (GD-1.5; Narishige, Tokyo, Japan) with a Flaming-Brown-type horizontal puller P-97 (Sutter Instruments, Novato, CA, USA). The electrode resistance ranged from 4.5 to 6.5 MΩ.

Astrocytes in the CA1 area were identified by SR101 fluorescence excited at 540 nm and emission detected with a high-pass filter (>570 nm). Whole cell recordings were made from SR101-positive cells with the pipette solution consisting of (in mm) 104 potassium methane sulphate, 36 KCl, 2 MgCl_2_, 10 Hepes-NaOH, 0.2 EGTA-KOH, 2.5 Na_2_ATP, 0.5 Na_2_GTP, pH 7.4, unless otherwise stated. The Cl^−^ concentration of the pipette solution was 40 mm, which was adjusted to previously reported physiological concentrations evaluated by gramicidin-perforated patch clamp (29 ± 3.2 mm (Bekar & Walz, 2002) or by radioactive Cl^−^ extrusion assay (30–50 mm (Kimelberg, 1981; Kettenmann *et al.* 1987). Astrocytic recordings were performed with a voltage clamp configuration held at –80 mV after evaluating the stabilized electrophysiological properties in a current clamp configuration. The typical classical astrocytes displayed a highly negative resting membrane potential (−85.4 ± 0.5 mV, *n*= 115), a low input resistance (31.6 ± 1.7 MΩ, *n*= 115, evaluated by current injection of −200 pA for 1 s), and a lack of voltage-gated sodium currents.

For GABA pressure application, GABA (1 mm or 50 μm) was added to the perfusing solution, and applied through a multi-barrelled pipette placed above a slice with a horizontal distance of 30–60 μm from the recording cell. The applied pressure was set at 20–40 kPa. To study GABA_A_ receptor antagonist-insensitive currents, recordings were made in the presence of 200 μm picrotoxin (PTX). To maintain the slice conditions under the continuous perfusion with PTX, which can induce seizure-like hyperexcitability, synaptic activity and neuronal firing were blocked with 1 μm tetrodotoxin and a Ca^2+^-free extracellular solution consisting of (in mm) 126 NaCl, 2.5 KCl, 1.25 NaH_2_PO_4_, 12.0 MgSO_4_, 26.0 NaHCO_3_ and 20.0 glucose, pH 7.4 when gassed with 95% O_2_/5% CO_2_. When voltage steps were applied to the recording astrocytes, 2 mm Ba^2+^ was added to the Ca^2+^-free extracellular solution to suppress the effects of voltage-gated K^+^ outward currents (Bekar & Walz, 2002).

In experiments with dual whole cell patch clamp recordings between an interneuron–astrocyte pair, recordings were performed in the stratum lacunosum-moleculare (SLM) or at the stratum radiatum (SR)–SLM border. In this region, a large proportion of interneurons make synapses within the SLM (Somogyi & Klausberger, 2005), including neuroglioform neurons whose axons branch close to the soma and produces an extremely dense axonal cloud (Price *et al.* 2005). An interneuron with a small, round soma identified by GFP fluorescence (excited at 470 nm with emission detected above 510 nm) and an astrocyte, usually 10–20 μm from the interneuron, were patch clamped with a current clamp (*I*_hold_= 0 pA) and a voltage clamp configuration, respectively. The internal solution for the interneurons consisted of (in mm) 135 potassium methane sulphate, 5 KCl, 2 MgCl_2_, 10 Hepes-NaOH, 0.2 EGTA-KOH, 2.5 Na_2_ATP, 0.5 Na_2_GTP, pH 7.4. A train of action potentials was evoked by repetitive current injection of +500 pA at 50 Hz for 2 s, and the single interneuron firing-induced currents were recorded from an adjacent astrocyte. The astrocytic current amplitude was evaluated as the difference between an average peak current for 50 ms around the visually identified peak and an average baseline for 1 s to reduce the influence of current noise.

Spontaneous IPSCs were recorded in a CA1 pyramidal neuron under the voltage clamp configuration held at −60 mV. In this recording, 6-cyano-7-nitroquinoxaline-2,3-dione (CNQX, 20 μm), d-(–)-2-amino-5-phosphonopentanoic acid (d-AP5, 50 μm) and CGP55845 (3 μm) were applied in the ACSF to block synaptic currents other than GABA_A_ receptor-mediated currents. The patch pipette solution consisted of (in mm): 130 CsCl, 1 CaCl_2_, 2 MgCl_2_, 10 Hepes-NaOH, 0.2 EGTA-KOH, 2.5 Na_2_ATP and 0.5 Na_2_GTP.

To analyse activity-dependent shifts in the reversal potential of neuronal IPSCs (*E*_IPSC_), whole cell voltage clamp recordings were made from a CA1 pyramidal neuron with the internal solution consisting of (in mm) 110 potassium methane sulphate, 2 KCl, 1 MgCl_2_, 10 Hepes-NaOH, 0.2 EGTA-KOH, 4 magnesium ATP, 20 K_2_-creatine phosphatase, 5 QX314 with 50 units of creatine phosphokinase, pH 7.4. In the presence of CNQX (20 μm), d-AP5 (50 μm) and CGP55845 (3 μm), tetanus stimulation (100 times at 200–400 pA, 200 μs, 50 Hz) at 1 min intervals was delivered at each holding potential, varying from −30 to −80 mV (10 mV decrements) through a monopolar glass pipette (filled with ACSF), which was placed in the SLM. The current–voltage (*I–V*) relationship after each stimulus was plotted to calculate the *E*_IPSC_. Current amplitudes were determined by the average of the *E*_IPSC_ recorded 150–180 ms after each stimulus.

The series resistance was usually below 25 MΩ and was compensated by 60–70% in most recordings. In astrocytes, recordings were rejected when base currents changed by more than 200 pA during the experiment. The series resistance of the pyramidal neurons was monitored throughout the experiments with voltage steps, and recordings were eliminated when series resistance changed by more than 20%. Reported voltage values were corrected by liquid junction potentials of 14.5 mV for astrocytes and 20.2 mV for pyramidal neurons. Tetrodotoxin and octanol were purchased from Wako, Tokyo, Japan; CNQX, d-AP5, CGP55845, SNAP5114, carbenoxolone and SKF89976a were bought from Tocris, Bristol, UK; and PTX, bicuculline methiodide (BIC) and NO711 were obtained from Sigma-Aldrich, St. Louis, MO, USA.

### Cl^−^ imaging

Astrocytic Cl**^−^** imaging with 6-methoxy-*N*-ethylquinolinium iodide (MEQ; Molecular Probes, Eugene, OR, USA) in combination with whole cell patch clamp recording was conducted as previously described (Isomura *et al.* 2003). MEQ (0.5 mm) was dissolved in the internal solution and delivered to the astrocytes through a patch pipette. MEQ fluorescence was excited between 340 and 380 nm, and its emissions, filtered at 435–485 nm, were captured by a CCD camera (Fukuda *et al.* 1998). Changes in fluorescence were recorded by placing regions of interest over part of the patch clamped cell soma (5.6 × 5.6 μm), using the image acquisition system AquaCosmos 2.6 (Hamamatsu Photonics). For analysis, background fluorescence was subtracted and photobleaching of the indicator was corrected linearly according to the slope for 2 s before GABA application. The index Δ*F/F* was used to estimate the relative change in [Cl^−^]_i_; *F* is the averaged fluorescence intensity obtained for 2 s before GABA application, and Δ*F* is the increase in fluorescence intensity excited at a given time. Quenching of the MEQ fluorescence, corresponding to a Cl^−^ increase, is expressed as a negative value in this index. Data were fitted to a two-dimensional local regression model using Kyplot software 4.0 (KyensLab, Inc., Tokyo, Japan). An increase in fluorescence intensity over baseline fluorescence (*ΔF/F*) indicated a relative decrease in [Cl^−^]_i_.

### Local GABA application using photo-uncaging systems

Laser photolysis of α-carboxy-2-nitrobenzyl-caged GABA (Invitrogen, Grand Island, NY, USA) was performed using a Micropoint laser system (Photonic Instruments, St. Charles, IL, USA). In the presence of CGP55845 (3 μm), whole cell voltage clamp recordings were made in a SLM astrocyte with an internal solution containing 0.2% Lucifer yellow. Caged GABA (2.5 mm) was dissolved in phosphate-buffered saline and delivered at a flow rate of 1 μl min^−1^ close to the patch clamped cell using a microdialysis syringe pump (Univentor, Zejtun, Malta) and a syringe with an inner tip diameter of 100 μm. A single pulsed nitrogen laser beam (365 nm wavelength, 600 ps pulse duration; KEN-3010; Ushio, Tokyo, Japan) was delivered to the slice via a quartz optical fibre through a 40× water-immersion objective (Fluor, 40×, NA 0.8; Nikon) to trigger local photolysis of caged GABA. The UV beam formed an uncaging spot approximately 5 μm in diameter at half-maximal intensity, which was focused on to the edge of the somatic region of a patch clamped astrocyte, or the edge of the soma of a surrounding astrocyte, with or without dye coupling. Currents induced by local GABA photolysis to each area were evaluated to analyse the spatial distribution of the GABA responding region in SLM astrocytes.

Local GABA photolysis-induced currents under the suppression of K^+^ channels was evaluated by means of dual whole cell patch clamp recordings from a pair of astrocytes approximately 50 μm apart. BaCl_2_ (2 mm) was added to low Cl^−^ ACSF consisting of (in mm) 126 C_2_H_5_NaO_4_S (sodium isethionate), 2.5 KCl, 1.25 NaH_2_PO_4_, 5.0 MgSO_4_, 26.0 NaHCO_3_ and 20.0 glucose, pH 7.4 when gassed with 95% O_2_/5% CO_2_. In these recordings, the voltage was clamped near the resting membrane potentials (−30 mV), which were depolarized by extracellular Ba^2+^ (−35 to −25 mV). To increase the driving force for Cl^−^, we used low Cl^−^ ACSF. The patch pipette solution consisted of (in mm): 130 CsCl, 1 CaCl_2_, 2 MgCl_2_, 10 Hepes-NaOH, 0.2 EGTA-KOH, 2.5 Na_2_ATP and 0.5 Na_2_GTP. Electrical coupling was confirmed by evaluating a coupling coefficient for current injection as the ratio of voltage deflection in the non-injected cell (referred to as the receiving cell) to that in the current injected cell (referred to as targeted cell) under the current clamp configuration. Then, voltage was clamped at −30 mV in both cells to record the currents induced by GABA photolysis to the edge of the targeted cell.

### Data acquisition and analysis

Membrane currents or membrane potentials were recorded using an Axopatch 200B or MultiClamp 700B amplifier (Molecular Devices, Sunnyvale, CA, USA), and signals were low-pass filtered at 2 kHz and digitized at 5–10 kHz by means of a Digidata 1332A data acquisition system (Molecular Devices). Pulse generation and data collection were performed using pCLAMP 9 or 10 software (Molecular Devices) and analysed offline using Clampfit 9 or 10 (Molecular Devices). The amplitudes of the GABAergic neuron firing-induced astrocytic currents were calculated by averaging over 50 ms around the peak. The time courses of these currents were calculated after low-pass filtering at 300 Hz. For clarity, recordings with peak current amplitudes below 6.0 pA were rejected for time course analysis.

All data are presented as the means ±s.e.m. One-way ANOVA followed by Dunnett's *post hoc* test or two-tailed Student's *t* test was used for statistical analysis. Two-way repeated measures ANOVA followed by Bonferroni's post-test was applied to analyse the *E*_IPSC_ increment after every five tetanic stimulations.

## Results

### GABA application-induced currents are mediated by both GABA_A_ receptors and mGAT4 in hippocampal CA1 astrocytes

**Figure 1:**
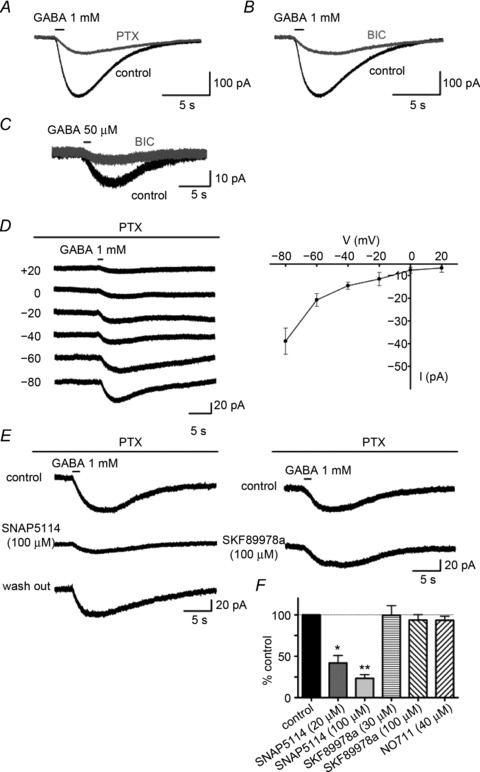
GABA application induces GABA_A_ receptor-mediated and mGAT4-mediated currents in CA1 astrocytes *A*–*C*, GABA-induced currents in control CA1 astrocytes (black traces) and in the presence of the GABA_A_ receptor antagonist (grey trace). GABA (1 mm)-evoked currents were reduced but not completely blocked by 200 μm PTX (*A*) or 20 μm BIC (*B*). BIC (20 μm) had a similar effect on the inward currents as that evoked by a lower concentration of GABA (50 μm) (*C*). *D*, voltage dependence of PTX-insensitive GABA currents. The left panel shows representative PTX-insensitive currents recorded at different holding potentials in the presence of 200 μm PTX and 2 mm Ba^2+^. The right panel shows an *I–V* plot constructed from five independent experiments. The plot never crossed over to positive current amplitudes above the calculated *E*_Cl_ (−31 mV). *E*, response of PTX-insensitive GABA currents to GAT inhibitors. SNAP5114, a non-competitive mGAT4 inhibitor, reversibly reduced the PTX-insensitive currents in a dose-dependent manner, *P < 0.05, **P < 0.01 (left panel). In contrast, SKF89978a, a non-competitive GAT1 inhibitor, did not alter the PTX-insensitive currents (right panel). *F*, the bar graph summarizes the effects of GAT inhibitors on the PTX-insensitive currents. BIC, bicuculline methiodide; PTX, picrotoxin.

To clarify the constituents of GABA-induced currents and Cl^−^ flux in CA1 astrocytes, we first examined the response to GABA application using whole cell voltage clamp recordings. Under physiological conditions (holding potential of −80 mV, 40 mm[Cl^−^]_i_), pressure application of GABA (1 mm, 1 s) evoked inward currents of 81.9 ± 8.1 pA (*n*= 30) in CA1 astrocytes. Bath application of PTX (200 μm) and BIC (20 μm) reduced the inward currents to a similar extent (PTX, 68.8 ± 2.9% reduction, *n*= 24; BIC, 70.2 ± 4.0% reduction, *n*= 6), but residual currents remained in all conditions (Fig. 1*A* and *B*). The same results were obtained with a lower GABA concentration (50 μm; from 16.4 ± 3.1 pA to 4.1 ± 0.4 pA, with a 73.3 ± 2.1% reduction by BIC, *n*= 5; Fig. 1*C*), indicating that incomplete antagonism did not affect residual currents. As the amplitude of GABA-evoked currents and PTX-insensitive component showed no significant differences from the amplitudes of recordings obtained from the SR and SLM (data not shown), data from both layers were pooled throughout the GABA application experiments. The *I–V* relationship of the PTX-insensitive, GABA-evoked currents did not reverse to positive membrane potentials at a calculated equilibrium potential for Cl^−^ (*E*_Cl_) of −31 mV (Fig. 1*D*).

We tested the effects of subtype-specific GAT inhibitors on the PTX-insensitive GABA currents (Barakat & Bordey, 2002; Kinney & Spain, 2002). SNAP5114, a non-substrate mouse GAT4 (mGAT4; Slc6a11) selective inhibitor, significantly and reversibly reduced the PTX-insensitive GABA currents in a dose-dependent manner (58.2 ± 9.1% reduction with 20 μm SNAP5114, *n*= 6, *P* < 0.05; 76.7 ± 4.5% reduction with 100 μm SNAP5114, *n*= 5, *P* < 0.01; Fig. 1*E*). In contrast, non-substrate GAT1 (Slc6a1)-selective inhibitors (SKF89976a or NO711) did not significantly reduce the PTX-insensitive GABA currents (6.4 ± 6.6% reduction by 100 μm SKF89976a, *n*= 5; 6.6 ± 4.9% reduction by 40 μm NO711, *n*= 4; Fig. 1*E*). These results demonstrate that GABA-induced currents are mediated by GABA_A_ receptors and mGAT4 in CA1 astrocytes.

### GABA application induces bidirectional Cl^−^ flux via GABA_A_ receptors and mGAT4 in CA1 astrocytes

**Figure 2:**
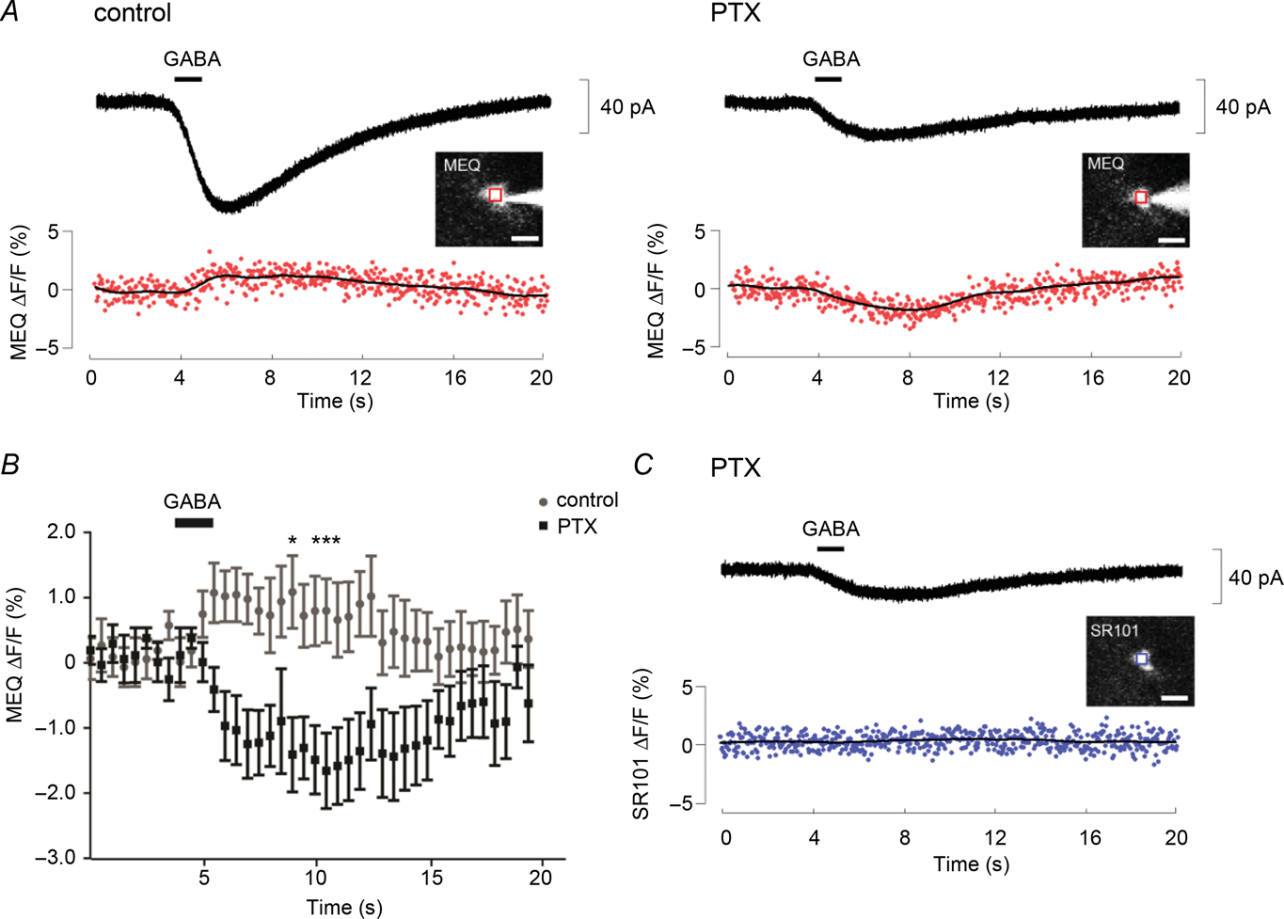
Optical imaging of Cl^−^ alterations induced by GABA application to CA1 astrocytes *A*, simultaneous recordings of currents (top) and MEQ fluorescence changes (bottom) evoked by 1 mm GABA application in controls (left panel) or in the presence of 200 μm PTX (right panel). The insets are fluorescent images of a recorded astrocyte to which MEQ was delivered through the patch electrode. The region of interest placed on the cell soma is indicated by a red square. The GABA-mediated increase in MEQ fluorescence became inverted after perfusion with PTX; both changes were synchronous with the GABA-evoked inward currents. *B*, average time course of MEQ fluorescence changes before (grey) and after (black) PTX perfusion (*n*= 8). Data at each time point were calculated by averaging for 500 ms on each recording. *P < 0.05 by two-way repeated measure ANOVA. Quenching of the MEQ fluorescence, corresponding to a Cl^−^ increase, is expressed as a negative value. *C*, SR101 fluorescence of the cell shown in (*A*) was not altered by GABA application upon perfusion with PTX. The inset shows a fluorescent image of the cell preloaded with SR101. The region of interest placed on the cell soma is indicated by a blue square. Scale bars: 10 μm. MEQ, 6-methoxy-*N*-ethylquinolinium iodide; PTX, picrotoxin.

Activation of GABA_A_ receptors produces Cl^−^ efflux in CA1 astrocytes. It is possible that mGAT4 transfers Cl^−^ in the opposite, inward direction, because GATs take up GABA with two Na^+^ and one Cl^−^ (Kanner & Schuldiner, 1987). However, Cl^−^ influx via GATs has not been clearly illustrated *in situ*. Therefore, we analysed GABA-induced [Cl^−^]_i_ alterations using Cl^−^ imaging with the Cl^−^ indicator MEQ in combination with simultaneous whole cell voltage clamp recording. In the absence of PTX, GABA increased MEQ fluorescence (corresponding to a [Cl^−^]_i_ decrease) in association with the inward currents. In contrast, MEQ fluorescence decreased (i.e. [Cl^−^]_i_ increased) after PTX perfusion, accompanying the residual mGAT4 currents (Fig. 2*A* and *B*). The fluorescence signal in the same cell from SR101, which labels astrocytes, was not changed by GABA application (*n*= 7; Fig. 2*C*); therefore, the decrease in MEQ fluorescence was not affected by unrelated events such as cell swelling. These data signified that the [Cl^−^]_i_ increases in astrocytes were mediated by Cl^−^ co-transport with GABA via mGAT4. Contrastingly, in pyramidal neurons, GABA did not decrease MEQ fluorescence in the presence of PTX under similar activation protocols (Supplementary Fig. 1). Previous immunohistochemistry data showed that neuronal GAT1 is predominantly located in presynaptic GABAergic terminals (Radian *et al.* 1990; Heja *et al.* 2009). Taken together, GABA application predominantly induces Cl^−^ efflux via GABA_A_ receptors in CA1 astrocytes under physiological conditions, but is accompanied by a non-negligible Cl^−^ influx via mGAT4.

### A train of single interneuron firing induces GABA_A_ receptor-mediated currents in an adjacent astrocyte in the stratum lacunosum-moleculare layer

Astrocytic GABA receptors respond to spillover of GABA from the synaptic cleft; thus, the properties of synaptically activated responses would be different from the responses to GABA application. To examine the dynamics of perisynaptic astrocytes in GABAergic tripartite synapses, we employed dual whole cell patch clamp recordings from interneuron–astrocyte pairs using GAD67-GFP knock-in mice (Tamamaki *et al.* 2003).

**Figure 3:**
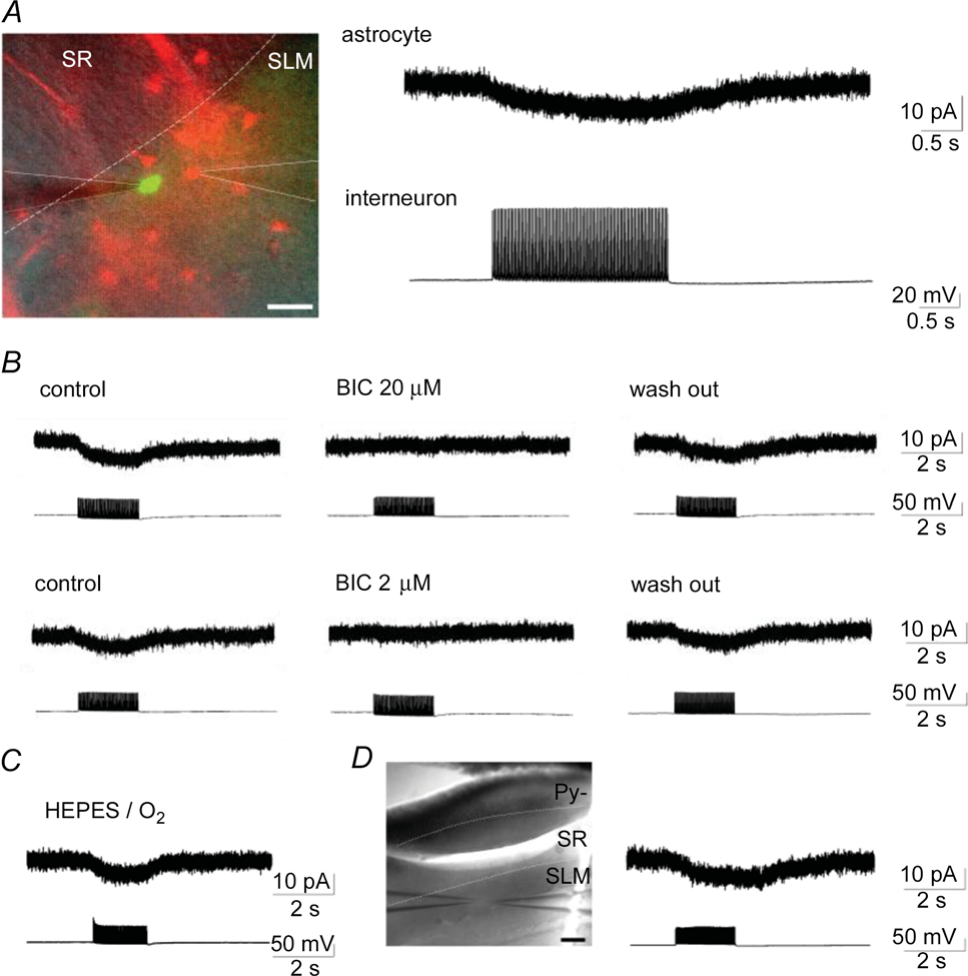
Trains of interneuron firing induce GABA_A_ receptor-mediated currents in an adjacent astrocyte in the SLM *A*, representative dual whole-cell patch clamp recording from SLM (dotted line) interneuron–astrocyte pair using *GAD67*-*GFP* knock-in mice. The left panel shows an overlay image of GFP (green) and SR101 (red) fluorescence and an infrared differential interference contrast image at the border of the SR and the SLM. The two patch electrodes in the SLM, emphasized with continuous lines, were placed on a close interneuron–astrocyte pair identified by GFP and SR101 fluorescence, respectively. Scale bar: 20 μm. Trains of interneuron firing initiated by repetitive current injection (right bottom) induced inward currents in the astrocyte, which we labelled as inhibitory synapse-driven astrocytic currents, *I*_inh-astro_ (right top); the arrangement is the same in subsequent figures. *B*, effects of BIC on *I*_inh-astro_. At 20 μm (top panel) or at much lower concentrations (2 μm; bottom panel), BIC completely and reversibly blocked the inward currents. *C*, *I*_inh-astro_ recorded in Hepes-buffered extracellular solution was similar to that in HCO_3_^−^-buffered solution (*A* and *B*). (*D*) *I*_inh-astro_ recorded in isolated SLM. Dendrites of pyramidal neurons inserting into the SLM were isolated from the cell soma by transversely cutting a slice in the SR (left panel). Astrocytic currents were similarly induced by trains of interneuron firing in this slice (right panel). Scale bar: 100 μm. BIC, bicuculline methiodide; Py., pyramidal neurons; SLM, stratum lacunosum-moleculare; SR, stratum radiatum.

In the SLM or at the SR-SLM border, trains of interneuron firing (50 Hz, 2 s) evoked by repetitive current injection of 500 pA induced small but obvious inward currents in an adjacent astrocyte (10–20 μm away) in 30 of 34 pairs (Fig. 3*A*). The mean current amplitude (6.0 ± 0.4 pA, *n*= 30) was not altered when the astrocytes were recorded with a Cs^+^-based intracellular solution (5.5 ± 1.4 pA, *n*= 8). The time course of these currents was characterized by a gradual increase during presynaptic firing (10–90% rise time: 1.23 ± 0.06 s, *n*= 17) and a relatively faster decrease after the firing (90–10% decay time: 1.14 ± 0.13 s). These single interneuron firing-evoked currents were completely and reversibly blocked by 200 μm PTX or 20 μm BIC (from 6.6 ± 1.2 to 0.3 ± 0.1 pA, *n*= 7, *P* < 0.01, using pooled data from PTX (*n*= 3) and BIC (*n*= 4); Fig. 3*B*, top panel). In addition, the currents were similarly blocked by a low 2 μm concentration of BIC (from 7.2 ± 1.7 to 0.4 ± 0.1 pA, *n*= 4, *P* < 0.05; Fig. 3*B*, bottom panel), a concentration that could only partially block the spontaneous IPSCs in CA1 pyramidal neurons (Supplementary Fig. 2). BIC antagonism of the GABA response is competitive, indicating that the single interneuron firing-evoked currents were mediated by astrocytic GABA_A_ receptors that responded to the low concentration of GABA spilt over from the synaptic clefts.

Postsynaptic GABA_A_ receptor-mediated HCO_3_^−^ conductance was previously shown to elevate [K^+^]_o_ (Voipio & Kaila, 2000), which might cause the BIC-sensitive astrocytic inward currents. However, a train of interneuron firing similarly induced the astrocytic currents in HCO_3_^−^-free, Hepes-buffered extracellular solution (5.2 ± 0.6 pA, *n*= 3; Fig. 3*C*). Moreover, the astrocytic currents could be recorded in the isolated SLM, by cutting a slice in the SR (5.8 ± 2.0 pA, *n*= 4; Fig. 3*D*). As isolated dendrites of CA1 pyramidal neurons were shown to lack IPSCs (Masukawa & Prince, 1984), these findings indicate that the postsynaptic component did not mediate the interneuron firing-induced currents in astrocytes. Thus, we termed these currents inhibitory synapse-driven astrocytic currents (*I*_inh-astro_). *I*_inh-astro_ were also recorded in wild-type mice and their amplitudes (5.5 ± 1.0 pA, *n*= 5) were comparable to that of GAD67-GFP knock-in mice. These data indicate that the heterozygous mutation of the GAD67 gene does not compromise signal transmission between inhibitory neurons to astrocytes, consistent with the previous report showing unaltered brain GABA content in GAD67-GFP knock-in mice (Tamamaki *et al.* 2003).

### GABA_A_ receptors are localized closer to the synaptic clefts than are mGAT4

In contrast to the response to GABA application, *I*_inh-astro_ did not contain mGAT4 currents. Although the *I*_inh-astro_ amplitude was much smaller than that of the 1 mm GABA-induced currents, the difference could not be explained by differences in their pharmacological affinity for GABA, because the proportion of BIC-insensitive currents induced by a lower concentration of GABA (50 μm) was equal to those induced by 1 mm GABA application (approximately a quarter, Fig. 1*A*). Therefore, the difference may be attributable to the distinct localization of GABA_A_ receptors and mGAT4.

**Figure 4:**
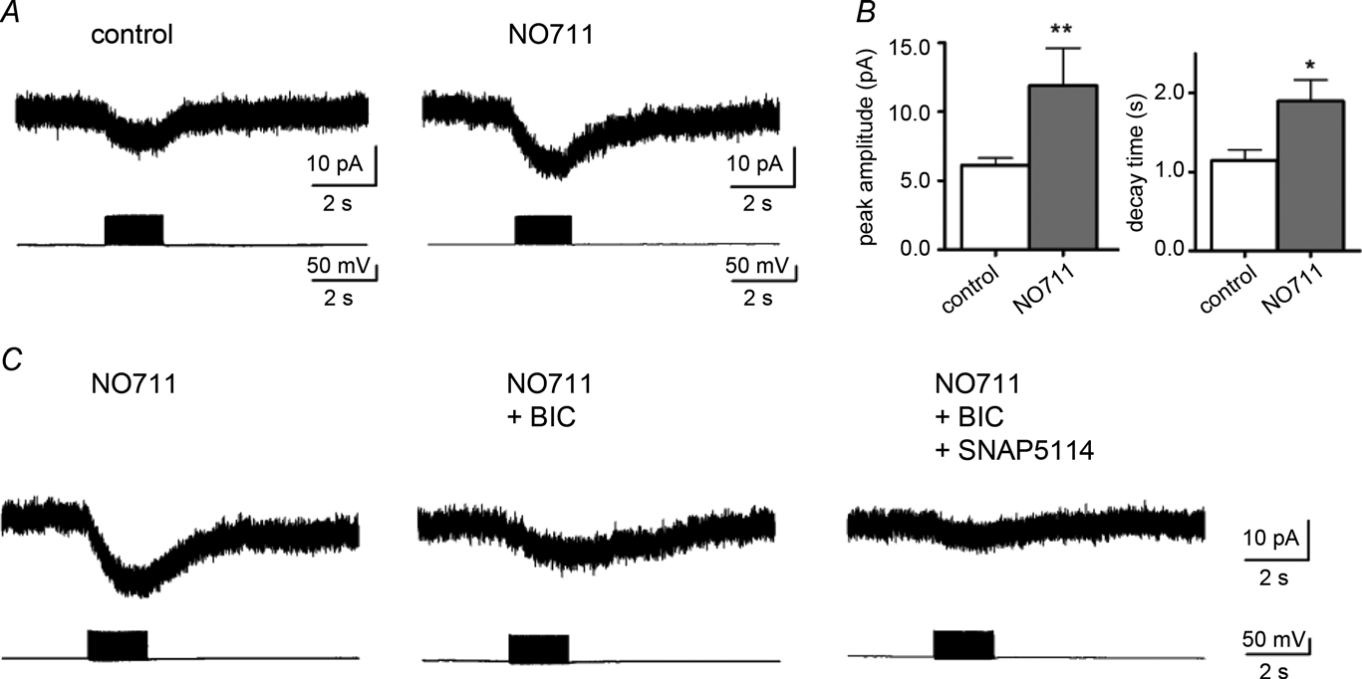
Blocking neuronal GAT1 reveals firing-induced astrocytic mGAT4 currents *A*, representative trace of *I*_inh-astro_ in a slice preincubated with 20 μm of the GAT1 inhibitor, NO711 (right panel) and in a control slice (left panel). *B*, the peak current amplitude and decay time were significantly increased in NO711-treated slices, *P < 0.05; **P < 0.01. *C*, *I*_inh-astro_ enhanced by NO711 (left panel) was reduced but not completely blocked by 20 μm BIC (middle panel). The residual currents were sensitive to additional application of the mGAT4 inhibitor, SNAP5114 (right panel). BIC, bicuculline methiodide.

To investigate this, we analysed *I*_inh-astro_ in slices preincubated with NO711 (20 μm), which inhibits neuronal GABA uptake. The peak current amplitude (11.9 ± 2.7 pA, *n*= 6) and the 90–10% decay time (1.90 ± 0.27 s, *n*= 6) of the *I*_inh-astro_ under GAT1 blockage were significantly greater than those of controls (current amplitude, 5.5 ± 0.3 pA, *n*= 10, *P* < 0.01; decay time, 1.08 ± 0.13 s, *n*= 7, *P* < 0.05; Fig. 4*A* and *B*). These data indicated that the released GABA was largely taken up by neuronal GAT1 and only GABA spillover could activate astrocytic GABA_A_ receptors. In the presence of NO711 (40 μm), BIC (20 μm)-insensitive currents were recorded in the *I*_inh-astro_. Most of the residual currents were mGAT4 currents because they were significantly reduced by additional application of 20 μm SNAP5114, from 4.9 ± 1.1 to 1.7 ± 0.4 pA (*n*= 6, *P* < 0.05, Fig. 4*C*). Thus, mGAT4 currents evoked by single interneuron firing were recorded only when neuronal GABA uptake was inhibited, indicating that the spillover GABA was predominantly taken up by neuronal GAT1. We could not rule out a possibility of a difference in affinity for GABA between GABA_A_ receptor and mGAT4. However, there are no reports showing the different densities between GABA_A_ receptors and mGAT4 in astrocytic processes, and neuronal GAT1 expression is predominantly localized to the axon terminals (Radian *et al.* 1990). The above results suggest that GABA_A_ receptors on astrocytic processes could be located closer to the synaptic cleft than the mGAT4 proteins.

### Gap junction conductance helps maintain interneuron firing-induced GABA_A_ receptor currents in stratum lacunosum-moleculare astrocytes

**Figure 5:**
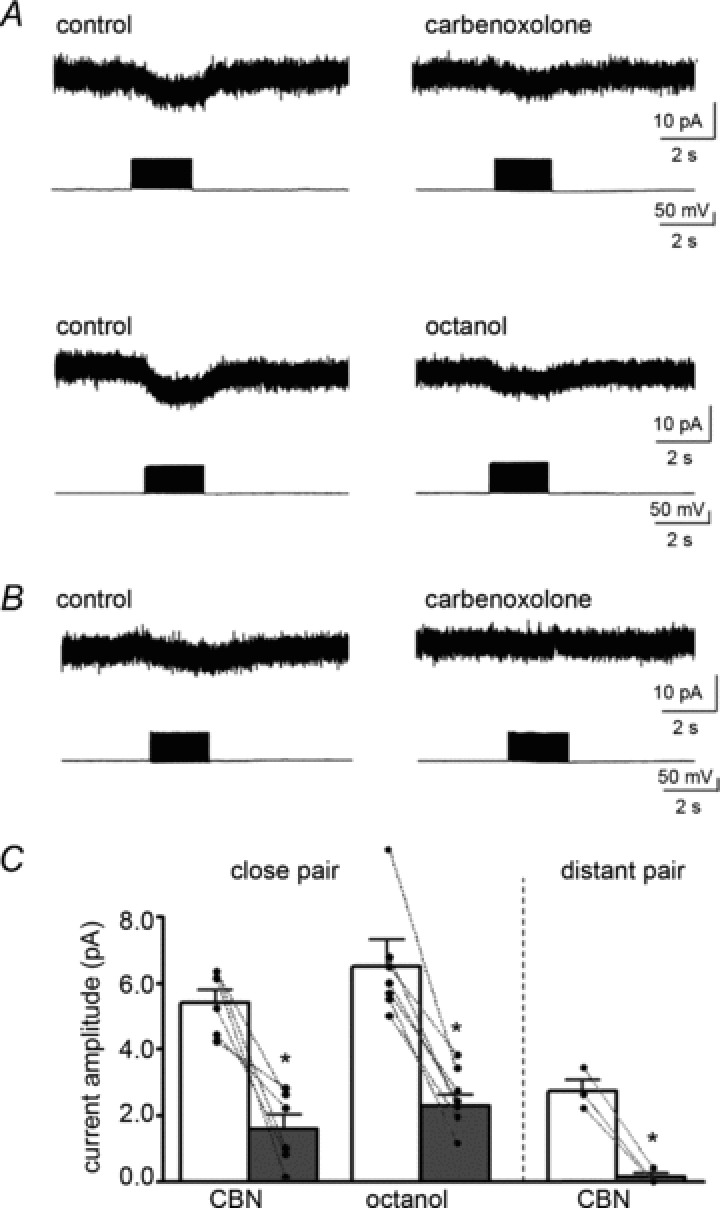
Gap junction inhibitors reduce *I*_inh-astro_ *A*, the effects of gap junction inhibitors on *I*_inh-astro_ in a close interneuron–astrocyte pair (10–20 μm apart). *I*_inh-astro_ was reduced by the application of CBN (500 μm, upper panels) or octanol (1 mm, lower panels). *B*, the effects of CBN (500 μm) on *I*_inh-astro_ in a distant interneuron–astrocyte pair (40–60 μm apart). The currents were smaller than those of close pairs and were completely blocked by CBN (500 μm). *C*, the effects of gap junction inhibitors on *I*_inh-astro_ in close or distant pairs. *P < 0.05. CBN, carbenoxolone.

*I*_inh-astro_ was recorded in 89% (30 of 34) of close interneuron–astrocyte pairs (10–20 μm apart). For more distant pairs (40–60 μm apart), *I*_inh-astro_ could still be obtained in six of 15 pairs (40%), but their peak current amplitudes were significantly smaller than those of close pairs (2.5 ± 0.2 pA, *n*= 6 *vs.* 6.0 ± 0.4 pA, *n*= 30, *P* < 0.05). The distance-related differences in current amplitude may suggest the propagation of GABA-mediated signals within the gap junction-coupled network. Therefore, we examined the involvement of gap junction communication in *I*_inh-astro_ by using the gap junction inhibitors, carbenoxolone (500 μm) or octanol (1 mm) (Supplementary Fig. 3, Juszczak & Swiergiel, 2009). Bath application of carbenoxolone (500 μm) or octanol (1 mm) significantly increased the input resistance of astrocytes (carbenoxolone, from 34.6 ± 4.5 to 54.9 ± 9.3 MΩ, *n*= 7, *P* < 0.05; octanol, from 39.8 ± 4.8 to 81.0 ± 13.3 MΩ, *n*= 7, *P* < 0.05), whereas the resting membrane potential was not affected by these agents (carbenoxolone, from −88.0 ± 2.3 to −84.1 ± 2.2 mV, *n*= 7, *P*= 0.24; octanol, from −84.0 ± 2.4 to −83.0 ± 2.9 mV, *n*= 7, *P*= 0.55) in line with previous uncoupling experiments (Wallraff *et al.* 2006). In this condition, *I*_inh-astro_ in close pairs was significantly reduced by carbenoxolone (from 5.4 ± 0.4 to 1.6 ± 0.4 pA, *n*= 6, *P* < 0.01) or octanol (from 6.5 ± 0.8 to 2.3 ± 0.3 pA, *n*= 7, *P* < 0.001) (Fig. 5*A* and *C*). The average reduction was approximately 65%, but this varied from pair to pair (33–98% reduction). The efficacy of gap junction inhibition correlated with the distance, because the smaller *I*_inh-astro_ in distant pairs was almost completely abolished by carbenoxolone (from 2.7 ± 0.3 to 0.1 ± 0.1 pA, *n*= 3, *P* < 0.01; Fig. 5*B* and *C*).

While interneurons in the SLM are also electrically coupled (Bushong *et al.* 2002; Zsiros & Maccaferri, 2005), it is unlikely that they contribute to the gap junction inhibitor-sensitive components of *I*_inh-astro_. Neuronal electrical synapses display marked low-pass filter properties, so that action potential trains barely propagate to postsynaptic interneurons (Zsiros & Maccaferri, 2005). Indeed, we did not observe action potential propagation during dual whole cell patch clamp recordings from electrically coupled interneuron pairs (Supplementary Fig. 4). These findings indicate that the observed astrocytic *I*_inh-astro_ contains current conductance via gap junctions from electrically coupled astrocytes.

**Figure 6:**
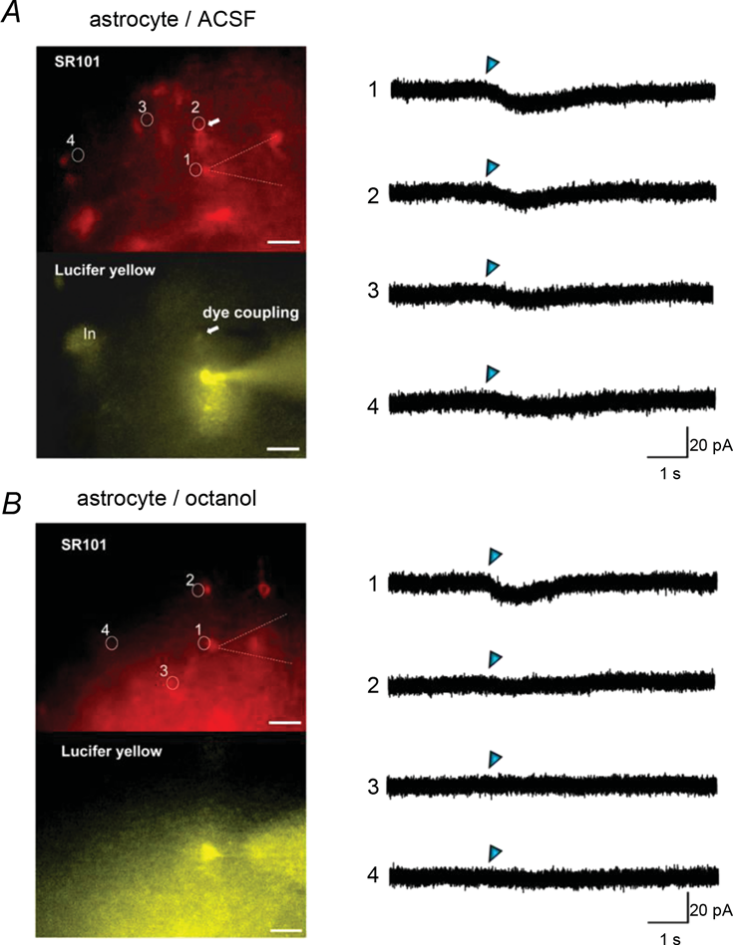
Local GABA application-induced currents propagate to electrically coupled astrocytes *A*, astrocytic responses to local GABA photolysis in the absence of gap junction inhibitors. The left upper panel is a SR101 fluorescence image. Numbered circles here and in (*B*) indicate uncaging spots, which correspond to the traces of uncaged GABA-induced currents in the right panel. Blue triangles indicate the time-points of a 0.9 ns uncaging flush. The patch pipette is indicated by a dashed line. The arrow indicates a dye-coupled astrocyte identified by Lucifer yellow fluorescence (left bottom panel) delivered through a patch pipette. (In) indicates an interneuron labelled by green fluorescent protein. Uncaged GABA-induced currents were recorded by photolysis, focusing on the edge of the patch clamped cell soma (1) or the edge of the dye-coupled cell soma (2). Smaller inward currents were recorded by photolysis on the distant cell somas without dye coupling (3, 4). *B*, these panels are the same as in (*A*), but in a slice preincubated with octanol (1 mm). Dye coupling was not observed. The GABA-responsive region was restricted to the vicinity of the recorded astrocyte. Scale bars: 20 μm. SR101, sulforhodamine 101.

To clarify the gap junction conductance of astrocytic GABA_A_ receptor-mediated currents, we evaluated the spatial profile of local GABA application to an electrically coupled astrocyte using a UV laser photo-uncaging system (Supplementary Fig. 5). Whole cell voltage clamp recordings were obtained in a SLM astrocyte, with the internal solution containing 0.2% Lucifer yellow to visualize dye-coupled astrocytes. One to three dye-coupled astrocytes were observed in all four slices in the absence of gap junction inhibitors. Local photolysis of caged GABA focused on to a dye-coupled astrocyte or a patch-clamped astrocyte induced inward currents in three of three slices (Fig. 6*A*). The ratio of current amplitude induced by photolysis on the dye-coupled astrocytes to that on the patch clamped astrocytes was 0.68 ± 0.12 (*n*= 4). Smaller inward currents were observed when photolysis was focused on to more distant astrocytes without dye coupling. As astrocytes occupy their space exclusively, and their processes avoid extensive interdigitation (Bushong *et al.* 2002), spatially distant GABA-responding regions indicate the existence of gap junctional communication. As expected, the GABA-responding region was restricted to the site of uncaging in slices incubated with octanol (*n*= 3; Fig. 6*B*).

**Figure 7:**
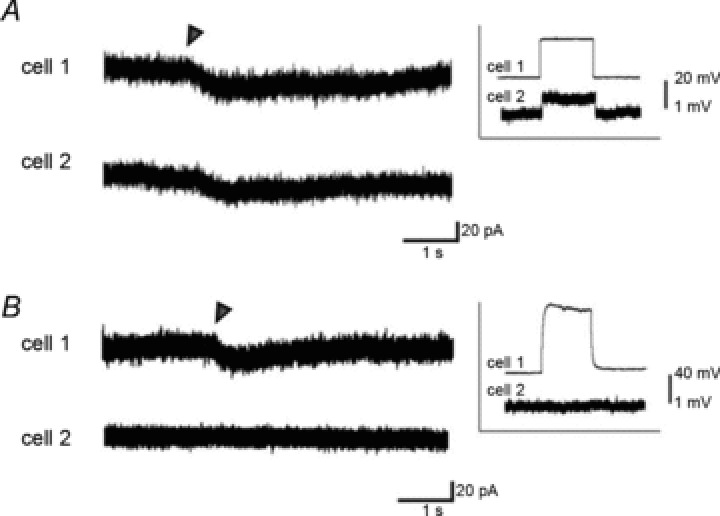
Local GABA application-induced currents conduct gap junction under the suppression of K^+^ channels *A*, representative voltage clamp recordings from a pair of stratum radiatum astrocytes 50 μm apart. K^+^ channels were blocked by extracellular Ba^2+^ and intracellular Cs^+^. Electrical coupling was confirmed by a voltage deflection in cell 2 (receiving cell) corresponding to a current injection (400 pA) to cell 1 (targeted cell) under the current clamp configuration (inset). Currents induced by local GABA photolysis (indicated by triangle) focused on the vicinity of soma of cell 1 were recorded not only in cell 1 but also in cell 2. *B*, traces are as in (*A*), but in a slice preincubated with octanol (1 mm). Pair of astrocytes electrically uncoupled as shown in the inset. GABA-photolysis-induced currents were observed only in cell 1.

GABA-induced depolarization might be conducted via gap junctions by an electrochemical gradient of other ions such as K^+^. Because of an overlap of excitation wavelength for caged GABA and MEQ, it was difficult to illustrate GABA-induced Cl^−^ flux via gap junctions by Cl^−^ imaging using MEQ. Thus, we examined whether the GABA-induced current could be conducted via gap junctions between a pair of astrocytes with equal holding potential and isometric ionic concentration under the suppression of astrocytic K^+^ currents. Dual whole cell voltage clamp recordings were performed between a pair of electrically coupled astrocytes in the SR approximately 50 μm apart in the presence of extracellular Ba^2+^ (2 mm). Recording astrocytes were loaded with CsCl (130 mm) via a patch pipette, and extracellular NaCl was replaced by sodium isethionate (calculated *E*_Cl_= 54.3 mV). In these conditions, the resting membrane potential was depolarized to −35.4 ± 4.5 mV (*n*= 6), caused by strong suppression of K^+^ channels, and the coupling coefficient evaluated by current injection to the targeted cells was 0.025 ± 0.003. Local GABA photolysis to the vicinity of the targeted cell soma induced inward currents not only in the target cell but also in the other cell (referred to as the receiving cell) with the voltage held at −30 mV (Fig. 7*A*, *n*= 3 pairs). The currents induced by indirect photolysis were not observed in a pair at the same distance in the presence of octanol (Fig. 7*B*, *n*= 2 pairs). These findings indicate that gap junctions conduct Cl^−^; thus, the [Cl^−^]_i_ is stably maintained in electrically coupled astrocytes. Therefore, extruded Cl^−^ via astrocytic GABA_A_ receptors is concomitantly complemented by an influx from other astrocytes via gap junctions, which might comprise the gap junction inhibitor-sensitive component of the inward *I*_inh-astro_.

### Gap junction inhibitors augment the activity-dependent depolarizing shifts in reversal potential for neuronal IPSCs

Homeostatic Cl^−^ conductance within the astrocytic network might play a vital role in the regulation of [Cl^−^]_o_ in GABAergic synapses by compensating for the Cl^−^ efflux via astrocytic GABA_A_ receptors. To test this hypothesis, we analysed the effects of gap junction inhibitors on *E*_IPSC_. Whole cell voltage clamp recordings were made from CA1 pyramidal neurons in the presence of CNQX (20 μm), d-AP5 (50 μm) and CGP55845 (3 μm). IPSCs evoked by tetanic stimulation (50 Hz, 2 s) to the SLM were recorded with various holding potentials from −30 to −80 mV with a 1 min interval, and the *E*_IPSC_ of GABA-mediated synaptic activation was evaluated.

**Figure 8:**
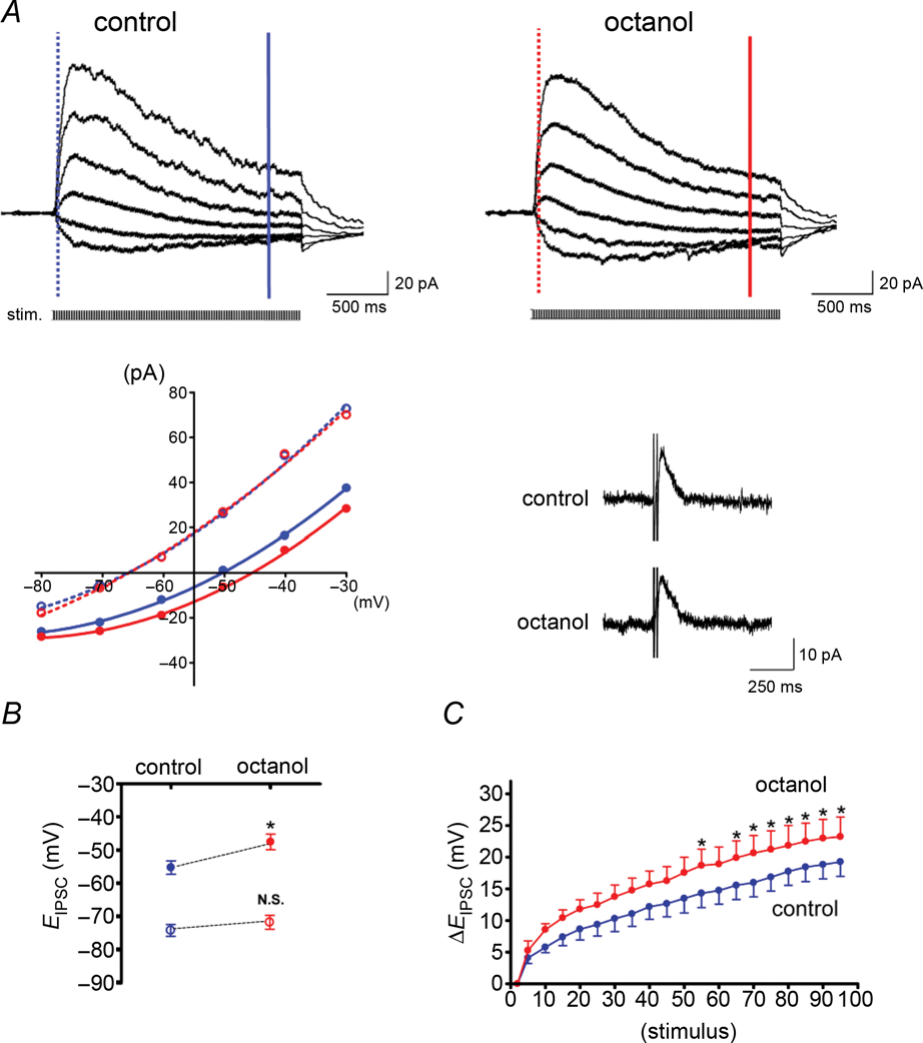
A gap junction inhibitor enhances activity-dependent depolarizing shifts in the *E*_IPSC_ *A*, recordings of GABA_A_ receptor-mediated postsynaptic currents in a CA1 pyramidal neuron evoked by tetanus stimulation (50 Hz, 2 s) to the SLM before and after perfusion of octanol (1 mm). Traces were low-pass filtered at 40 Hz to reduce stimulus artefacts. Traces were recorded with various holding potentials from −30 to −80 mV (10 mV decrement) with a 1 min interval and the baselines were superimposed. The *I–V* relationship after the second stimulus (early phase; indicated by the dashed lines and open circles) and the 90th stimulus (late phase; indicated by the continuous lines and filled circles) are plotted from each trace. Blue and red lines indicate before and after octanol perfusion, respectively. Note that octanol shifted the *E*_IPSC_ in the late phase of tetanus but not in the early phase. The inset shows IPSCs evoked by paired stimuli in the same cell, which were not altered by octanol. *B*, means ±s.e.m. of the *E*_IPSC_ in the early (○) and late phase (•). *C*, the change in *E*_IPSC_ from that of the second stimulus (Δ*E*_IPSC_) plotted after every five stimulations. Octanol significantly enhanced the Δ*E*_IPSC_ in the later phase of tetanus stimulation. *P < 0.05; N.S., not significant.

In controls, the *E*_IPSC_ of the 90th stimulus was significantly more positive than that of the second stimulus (−56.1 ± 1.7 *vs*. −74.9 ± 0.9 mV, *n*= 12, *P* < 0.0001). This activity-dependent depolarizing shift (Δ*E*_IPSC_=+18.6 ± 1.8 mV) resulted from a collapse of the Cl^−^ gradient because of intracellular Cl^−^ accumulation via GABA_A_ receptors (Staley *et al.* 1995; Staley & Proctor, 1999; Isomura *et al.* 2003). Subsequent application of octanol (1 mm) significantly enhanced Δ*E*_IPSC_ in the later phase of tetanus (on the 90th stimulus: from +19.2 ± 2.6 to +23.3 ± 3.3 mV, *n*= 6, *P* < 0.05; Fig. 8*A* and *C*). *E*_IPSC_ on the 90th stimulus (from −55.1 ± 2.0 to −47.4 ± 2.3 mV, *n*= 6, *P* < 0.01), but not on the second stimulus (from −72.1 ± 2.1 to −74.5 ± 1.8 mV, *P*= 0.12), shifted toward depolarization (Fig. 8*A* and *B*). The peak current amplitude and decay time of the IPSCs with single or paired pulse stimulation were unaffected by octanol (single pulse stimulation-evoked IPSCs at holding potentials of −40 mV, current amplitude: 96.8 ± 10.8 *vs*. 93.7 ± 10.2 pA, *n*= 7, *P*= 0.69; decay time: 163.1 ± 14.2 *vs*. 184.9 ± 24.6 ms, *n*= 7, *P*= 0.28). Further, octanol did not affect the input resistance of neurons (from 333.0 ± 38.4 MΩ to 368.3 ± 54.5 MΩ, *P*= 0.14) in contrast to that of astrocytes. Taken together with the result that *E*_IPSC_ in the early phase was not affected by octanol, these findings indicate that the effect of octanol on Δ*E*_IPSC_ is not caused by neuronal GABA_A_ receptor activation, which has been previously reported *in vitro* (Dildy-Mayfield *et al.* 1996).

We could not evaluate the effects of carbenoxolone because application of carbenoxolone (100 or 500 μm) significantly reduced evoked IPSCs (Supplementary Fig. 6), presumably due to its secondary effect of elevating action potential thresholds (Rouach *et al.* 2000). Other gap junction inhibitors, niflumic acid or meclofenamic acid, also significantly modulated neuronal IPSC (Supplementary Fig. 6) in accordance with previous reports (Sinkkonen *et al.* 2003; Coyne *et al.* 2007). Thus, we could analyse the effect of gap junction inhibitors on the intense inhibitory synapse transmission only by octanol. Nevertheless, these results suggest that the astrocytic network may play a significant physiological role in maintaining inhibitory synaptic transmission by spatially buffering the [Cl^−^]_o_ of the synaptic cleft to moderate a collapse of the Cl^−^ gradient in postsynaptic neurons (Supplementary Fig. 7).

## Discussion

The present study investigated functional properties of the astrocytic network on GABAergic tripartite synapses in the CA1 hippocampus, focusing on Cl^−^ homeodynamics in the neuron–astrocyte network. Our main findings are as follows (and illustrated in Supplementary Fig. 7): (1) with physiologically high [Cl^−^]_i_, GABA application induced bidirectional Cl^−^ flux in CA1 astrocytes, with Cl^−^ efflux via GABA_A_ receptors and Cl^−^ influx via mGAT4; (2) synaptic spillover of GABA predominantly induced Cl^−^ efflux from astrocytes presumably due to the localization of GABA_A_ receptors near the synaptic clefts; and (3) gap junctions between astrocytes conducted GABA-induced currents. Blockage of gap junctions enhanced the activity-dependent depolarizing shift in *E*_IPSC_, suggesting that net Cl^−^ extrusion from the gap junction-coupled astrocytic network might spatially buffer [Cl^−^]_o_ during intense activation of GABAergic synapses.

Previous immunochemical studies have shown that GAT expression is not cell-type specific (Rattray & Priestley, 1993; Minelli *et al.* 1995), or specific to the hippocampus (Ribak *et al.* 1996), e.g. GAT1-mediated currents were recorded in glial cells in rat cortex (Kinney & Spain, 2002) and cerebellum (Barakat & Bordey, 2002). In the present study, however, most functional GATs were mGAT4 in the CA1 hippocampal astrocytes. Similarly, a recent study showed that GAT1 and GAT3 (mGAT4) immunoreactivities were specifically localized on presynaptic neurons and astrocytes, respectively (Heja *et al.* 2009).

GAT currents are generated by net charge translocation attributable to ions co-transported with GABA, the stoichiometry of which is generally assumed to be 1 GABA:2 Na^+^:1 Cl^−^ (Kanner & Schuldiner, 1987). Although it is controversial whether Cl^−^ is transferred by GATs (Loo *et al.* 2000; Karakossian *et al.* 2005), in this study, the increases in [Cl^−^]_i_ were coincident with GABA-induced, PTX-insensitive currents and were demonstrated by Cl^−^ imaging using MEQ. These findings indicate that Cl^−^ was actually co-transported by mGAT4 *in situ*, as proven by tracer uptake measurements of GAT1 (Krause & Schwarz, 2005). The [Cl^−^]_i_ decrease without PTX was a consequence of Cl^−^ efflux via the GABA_A_ receptor overwhelming Cl^−^ influx via mGAT4.

mGAT4 currents were induced by single interneuron firing only when GAT1 was inhibited by NO711, indicating that the ambient GABA level was highly regulated by neuronal GAT1 when GABA release was incrementally increased by repetitive firing (Isaacson *et al.* 1993). This functional difference between neuronal GAT1 and astrocytic mGAT4 might be caused by their different expression intensity and/or different driving force because of the higher [Cl^−^]_i_ in astrocytes. In contrast to the synaptically released GABA-induced currents, the current induced by low-dose GABA application contained a BIC-insensitive component in the absence of NO711. The above results support our hypothesis that astrocytic GABA_A_ receptors could be localized more closely to the synaptic cleft than mGAT4. Therefore, Cl^−^ uptake via mGAT4 could play a complementary role in the astrocyte-mediated Cl^−^ homeostasis of GABAergic synapses. As we specifically evaluated astrocytic responses to single interneuron firing, the functional properties of mGAT4 under conditions of excessive neuronal network activity (Heja *et al.* 2009) remain to be elucidated.

Little is known about the functional properties of GABA spillover-induced electrical signalling in glial cells. Synaptically activated depolarization via GABA_A_ receptors has been demonstrated in hippocampal oligodendrocyte precursor cells (Lin & Bergles, 2004) and in stellate glial cells of the pituitary (Mudrickdonnon *et al.* 1993). However, depolarization was attributed to direct release of GABA on to glial cells rather than spillover from synaptic clefts. Focal fibre stimulation elevated [*K*^+^]_o_, which makes it difficult to evaluate the slow kinetic responses to GABA spillover in astrocytes with their low input resistance. In this study, by stimulating single GABAergic neurons, signal transmission from the GABAergic neuron to astrocytes (*I*_inh-astro_) could be directly evaluated without the contaminating effect of [K^+^]_o_ uptake currents.

Several lines of evidence illustrated that *I*_inh-astro_ was evoked by GABA spillover. First, *I*_inh-astro_ displayed much slower kinetics than the evoked IPSCs stimulated by the same configuration (50 Hz, 2 s; Fig. 8). Second, *I*_inh-astro_ was completely antagonized by a low dose of BIC, which did not abolish spontaneous IPSCs. Spillover of GABA was predominantly taken up by neuronal GAT1, not by glial mGAT4. This is in contrast to the modulation of neuronal tonic currents in the rat cortex, which requires inhibition of GAT3 as well as GAT1 (Keros & Hablitz, 2005). Because GAT1 expression clusters at presynaptic boutons (Chiu *et al.* 2002), our electrophysiological data indicated that the distribution of GABA_A_ receptors in astrocytic processes was peri- rather than extra-synaptic, as shown by electron microscopy of Bergmann glia (Riquelme *et al.* 2002). Thus, Cl^−^ efflux via astrocytic GABA_A_ receptors can contribute to the regulation of [Cl^−^]_o_ during GABAergic transmission (Kettenmann *et al.* 1984*a*; MacVicar *et al.* 1989).

Strong gap junction coupling between astrocytes probably mediates the coordinated action of coupled cells and equalizes their intracellular ion concentration (Rose & Ransom, 1997). In agreement with this hypothesis, spillover GABA-induced astrocytic signalling was propagated within the astrocytic network through gap junctions. Approximately two-thirds of *I*_inh-astro_ were blocked by two different gap junction inhibitors, although the gap junction inhibitor-sensitive currents might not be equivalent to Cl^−^ conductance via gap junctions. Because it is difficult to clamp the voltage of coupled astrocytes completely because of space clamp restrictions, these currents might be concomitant with the K^+^ currents that accompany GABA-induced depolarization. GABA_A_ receptor activation has been shown to inactivate A-type outward K^+^ currents (*I*_A_) in depolarized astrocytes (Bekar & Walz, 2002), which might comprise part of the gap junction inhibitor-sensitive *I*_inh-astro_. However, this is unlikely because the amplitude of *I*_inh-astro_ is not altered by intracellular Cs^+^ replacement, which blocks outward-rectifier K^+^ channels including *I_A_* (Banks & Pearce, 2000).

Alternatively, a slight GABA-mediated depolarization could be driven by voltage gradients across gap junctions with the carriage of K^+^. Further, GABA-mediated depolarization was counterbalanced by K^+^ efflux through passive K^+^ conductance, which sets the glial resting membrane potential close to the K^+^ equilibrium. In this view, the Cl^−^ conductance component via gap junctions in *I*_inh-astro_ might be underestimated. We illustrated that GABA-evoked currents were transmitted via gap junctions between equally voltage clamped astrocytes under the suppression of K^+^ channels and using 130 mm CsCl pipette solution in both cells. In this condition, a driving force for GABA-induced gap junctional conductance could be predominantly produced by the chemical gradient of Cl^−^ brought about by Cl^−^ efflux in the GABA-activated cell. Although Cl^−^ imaging is required to quantify a Cl^−^ conductance via gap junctions, these results may attest to the presence of a gap junctional Cl^−^ conductance induced by GABA_A_ receptor activation.

Astrocytes and interneurons are connected by gap junctions (Tamas *et al.* 2000; Zsiros & Maccaferri, 2005). Application of the gap junction inhibitor, octanol, augmented the activity-dependent depolarizing shifts in *E*_IPSC_. Because an electrical synapse in interneurons can regulate their synchronous firing (Tamas *et al.* 2000), octanol might decrease the total activation of interneurons in response to tetanus stimulation. In this case, the activity-dependent depolarizing shift should be decreased rather than increased. Therefore, octanol would have only a limited effect on properties of interneuron firings in our recording protocol. Octanol can alter GABA_A_ receptor permeability (Dildy-Mayfield *et al.* 1996). However, neither properties of IPSCs evoked by single pulse stimulation nor *E*_IPSC_ at the second stimulus of tetanic stimulation were affected by octanol. Thus, it is unlikely that octanol significantly modulated the properties of postsynaptic GABA_A_ receptors in this recording. Taken together, our results suggest that the astrocytic network may moderate the collapse of the inhibitory driving force for GABAergic synapses during intense GABAergic neuron firing.

Cl^−^ conductance via gap junctions could complement the [Cl^−^]_i_ decrease in astrocytes responding to GABA spillover by the GABA_A_ receptor, which would maintain Cl^−^ homeostasis by a siphon effect. As the GABA_A_ receptors of postsynaptic neurons and presynaptic GATs could take up Cl^−^ from the synaptic clefts, this astrocytic Cl^−^ conductance might spatially buffer the change in [Cl^−^]_o_ and maintain GABAergic synapse transmission. This idea is supported by a recent *in vivo* study using Cl^−^-sensitive electrodes that showed activity-dependent changes in [Cl^−^]_o_ (Kroeger *et al.* 2010).

As the initial [Cl^−^]_o_ is much higher than [Cl^−^]_i_, [Cl^−^]_o_ will have a minor effect on *E*_Cl_ changes, determined by the log of [Cl^−^]_o_/[Cl^−^]_i_. In our study, the Δ*E*_IPSC_ increment by gap junction closure was about 5 mV. In addition, because an increase in [K^+^]_o_ enhances the activity-dependent depolarizing shifts in GABAergic transmission by modulating the thermodynamics of the K^+^–Cl^−^ cotransporter (Staley & Proctor, 1999; DeFazio *et al.* 2000), blockage of glial spatial buffering of extracellular K^+^ by octanol could also be involved in the mechanism of depolarized *E*_IPSC_. Nevertheless, our present study suggests that [Cl^−^]_o_ in the nano-domain of synaptic clefts is much more dynamic than previously assumed (Kroeger *et al.* 2010). In this regard, astrocytes may act as a storage for Cl^−^, and could help maintain [Cl^−^]_o_ of the synaptic cleft. A limitation of this study is that we could analyse the functional significance of gap junction-coupled astrocytic network only by using one of the gap junction inhibitors because other drugs have shown to modulate neuronal GABAergic signalling (Supplementary Fig. 6). Further non-pharmacological analysis (e.g. genetic manipulation for astrocytic GABA_A_ receptors and/or gap junctions) is required to elucidate the molecular basis for the role of the astrocytic network in GABAergic transmission.

## Abbreviations

BIC: bicuculline methiodide
CNQX: 6-cyano-7-nitroquinoxaline-2,3-dione
d-AP5: d-(–)-2-amino-5-phosphonopentanoic acid
*E*_Cl_: equilibrium potential for Cl^−^
*E*_IPSC_: reversal potential of neuronal IPSCs
GAT: GABA transporter
GFP: green fluorescent protein
*I*_A_: A-type outward K^+^ current
*I*_inh-astro_: inhibitory synapse-driven astrocytic currents
MEQ: 6-methoxy-*N*-ethylquinolinium iodide
mGAT4: mouse GABA transporter 4
NKCC1: Na^+^/K^+^/2Cl^−^ cotransporter
PTX: picrotoxin
SLM: stratum lacunosum-moleculare
SR: stratum radiatum
SR101: sulforhodamine 101
