# Supplementary material for: Cl− homeodynamics in gap junction-coupled astrocytic networks on activation of GABAergic synapses
Source: J Physiol. 2013 Jun 3;591(Pt 16):3901–17. doi: 10.1113/jphysiol.2013.257162 (PMC3764636; doi:10.1113/jphysiol.2013.257162)
Supplement: Supplementary file 1 [file tjp0591-3901-SD1.pdf]

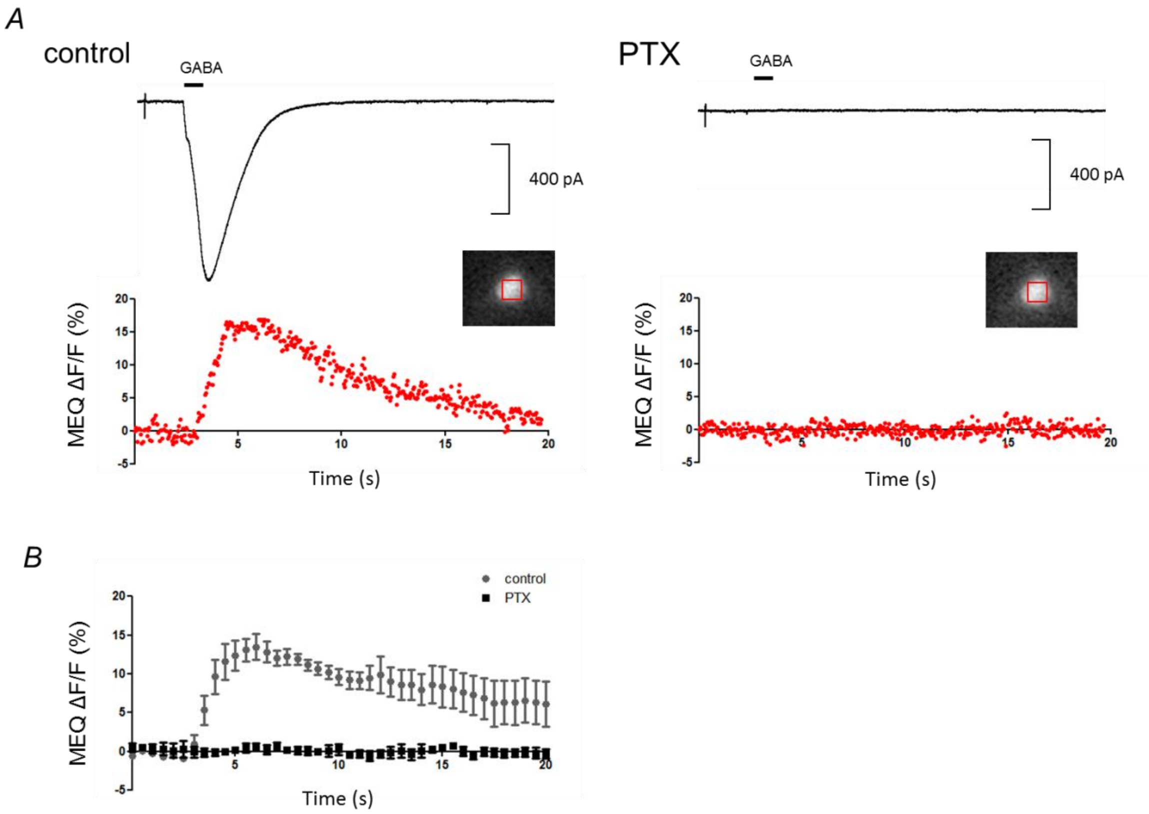


**Supplementary Figure 1.** Optical imaging of $Cl^-$ alterations induced by GABA application to CA1 pyramidal neuron. *A*, simultaneous recordings of currents (top) and MEQ fluorescence changes (bottom) evoked by 1 mM GABA application in controls (left panel) or in the presence of 200 μM PTX (right panel). The insets show fluorescence images of a recorded neuron to which MEQ was delivered through the patch electrode. The region of interest on the cell soma is indicated by a red square. In contrast to astrocytes, GABA did not evoke current or fluorescence changes under the presence of PTX. *B*, average time course of MEQ fluorescence changes before (grey) and after (black) PTX perfusion (n = 5). Data at each time point were calculated based on an average of 500 ms on each recording. *$P < 0.05$ by two way repeated measure ANOVA. Quenching of the MEQ fluorescence, corresponding to a $Cl^-$ increase, is expressed as a negative value.

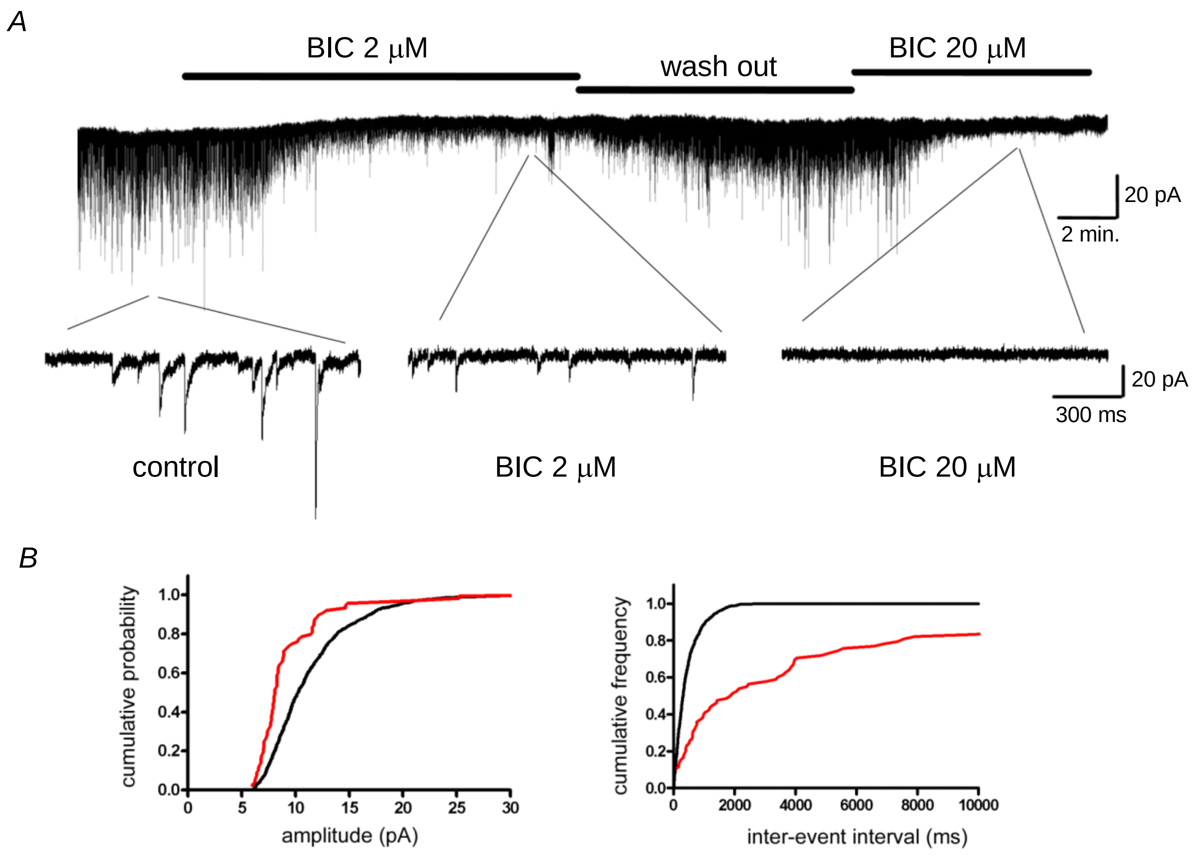


**Supplementary Figure 2**. BIC at 2 μM is insufficient for complete antagonism of neuronal IPSCs. *A*, representative spontaneous IPSCs (sIPSCs) recorded from a CA1 pyramidal neuron. BIC at 2 μM reduced, but did not completely block sIPSCs, while BIC at 20 μM completely abolished the sIPSCs. *B*, quantification of sIPSC amplitude and frequency under the presence of BIC at 2 μM (black line) or 20 μM (red line). Data are presented as cumulative probability plots of sIPSC amplitude (left panel) and inter-event intervals (right panel) evaluated from four neurons, indicating significant differences **(**$P < 0.0001$, Kolmogorov-Smirnov test).

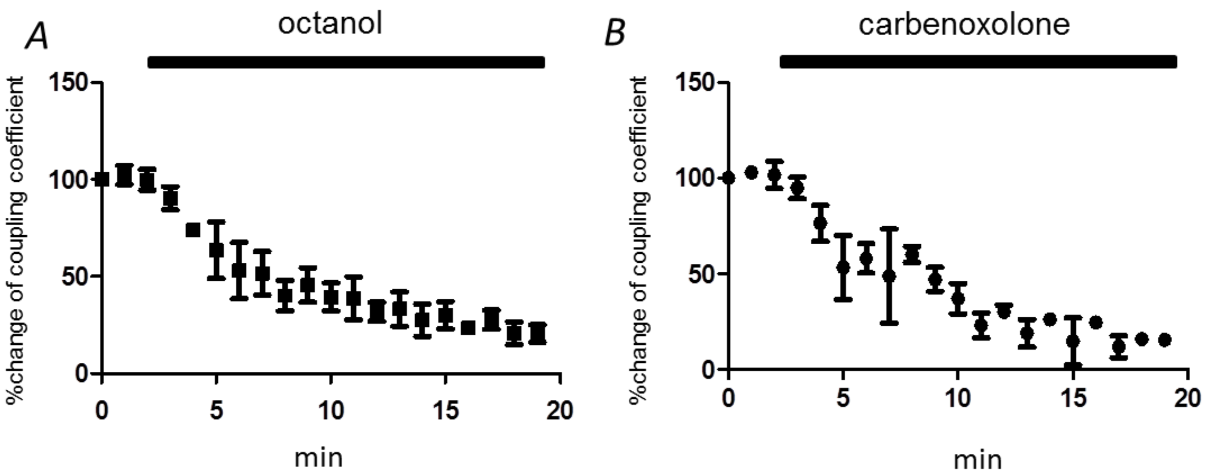


**Supplementary Figure 3**. Effects of gap junction inhibitors on electrical coupling in astrocytes. The strength of coupling between a pair of astrocytes was analysed by evaluating a coupling coefficient for current injection as the ratio of voltage deflection in the non-injected cell to that in the current injected cell under the current clamp configuration. The relative change in the coupling coefficient under the perfusion of (*A*) octanol (1 mM, n = 3) or (*B*) carbenoxolone (500 μM, n = 2) is plotted at one minute intervals.

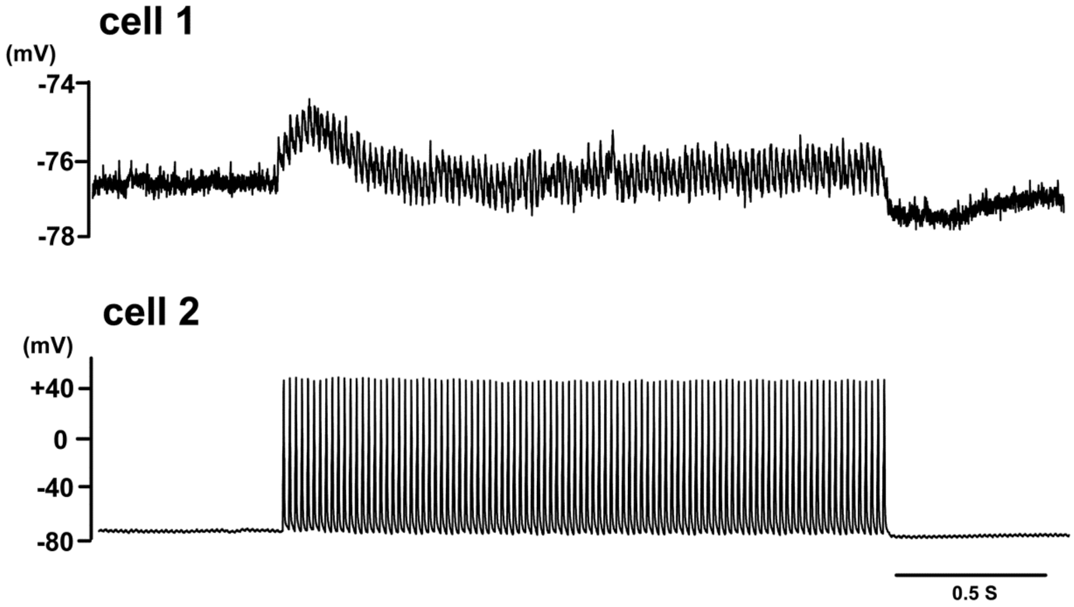


**Supplementary Figure 4**. A train of action potential firing does not propagate to the other interneurons *via* electrical coupling. Representative current clamp recordings from a pair of electrically coupled interneurons in the SLM are shown. A train of action potential firing was initiated by repetitive current injection in cell 2. Simultaneous current clamp recordings in cell 1 showed a slight depolarization up to a few millivolts, but never resulted in the propagation of action potentials.

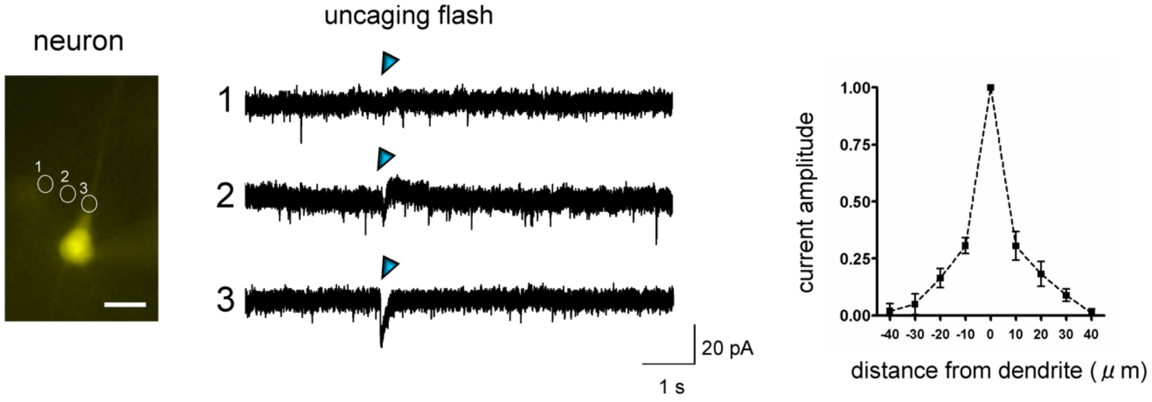


**Supplementary Figure 5.** Lateral resolution of GABA photolysis analysed by scanning the laser beam across a neuronal dendrite. The dendrite was identified by Lucifer yellow fluorescence (left panel, yellow) delivered through a patch pipette. Numbered circles indicate uncaging spots, which correspond to the traces of uncaged GABA-induced currents in the middle panel. Blue triangles indicate the time-points of a 0.9-ns uncaging flush. The right graph demonstrates the lateral resolution of GABA photolysis at 10-μm intervals (*n* = 5).

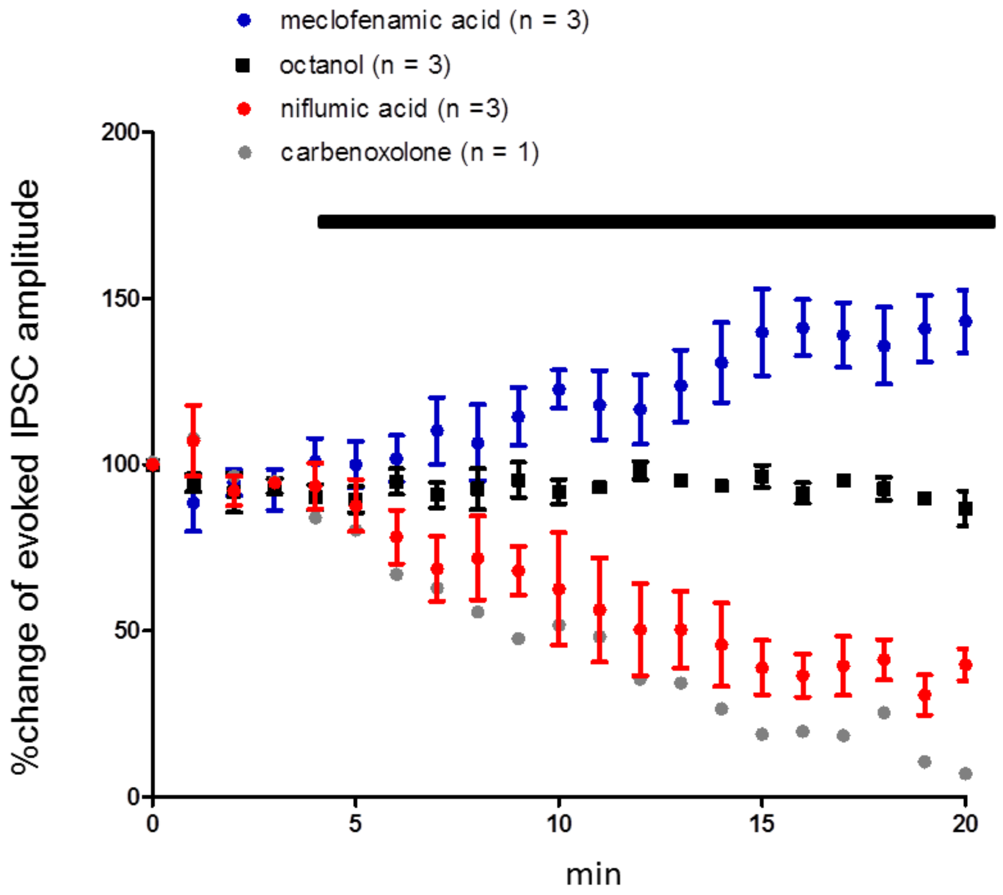


**Supplementary Figure 6.** Time course of the relative changes of evoked IPSC amplitude by gap junction inhibitors in CA1 pyramidal neuron

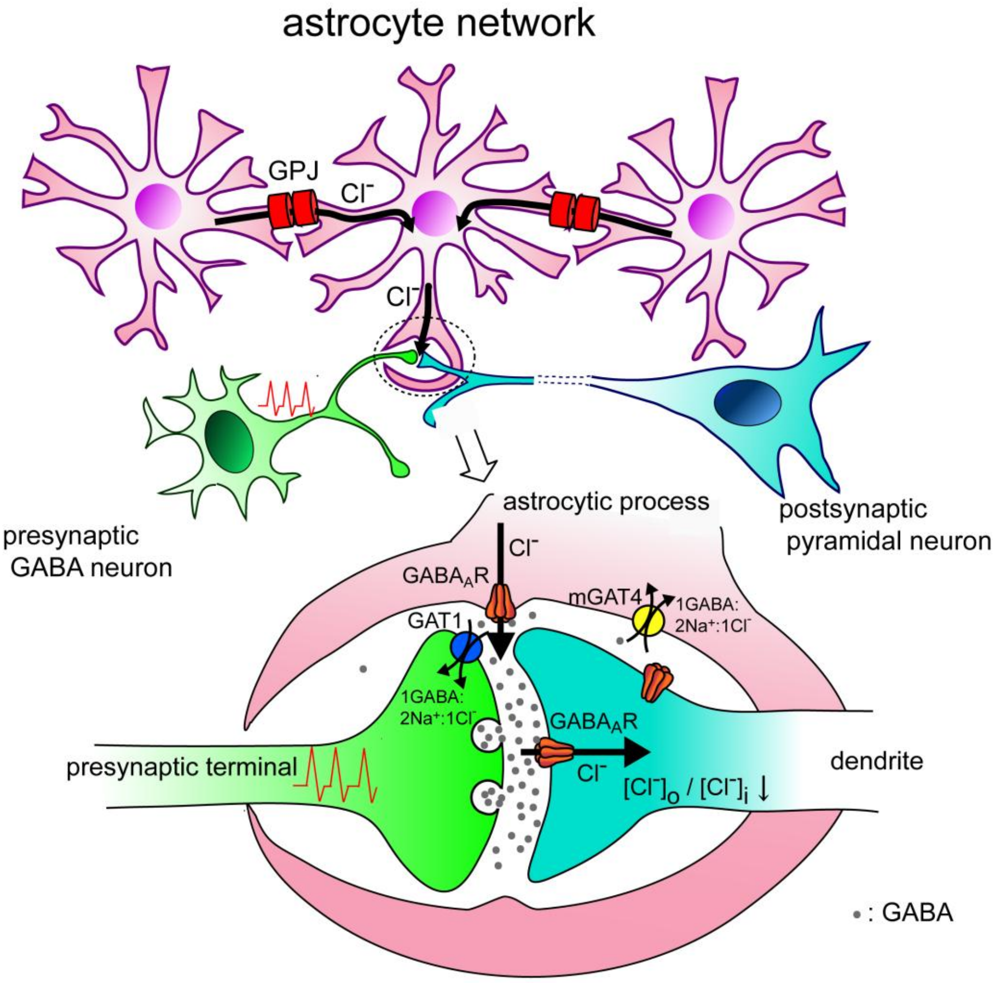


**Supplementary Figure 7.** Model of $Cl^-$ homeodynamics at a CA1 tripartite GABAergic synapse. An astrocytic process tightly envelops the GABAergic synapse and expresses both $GABA_A$ receptors ($GABA_AR$) and mGAT4. Spillover of GABA evoked by repetitive interneuron firing induces $Cl^-$ efflux *via* astrocytic $GABA_A$ receptors, which are localized near the synaptic cleft. The relatively distal localization of mGAT4 mediates $Cl^-$ influx in cooperation with GABA uptake, but its contribution may be negligible because diffusion of GABA is limited by neuronal GAT1. Astrocytic gap junctions (GPJ) tightly coordinate $[Cl^-]_i$ within coupled astrocytes. $GABA_AR$-induced $Cl^-$ efflux causes a siphon effect that induces simultaneous compensation by $Cl^-$ influx from non-activated astrocytes *via* GPJ, so that the driving force for $Cl^-$ efflux by astrocytic $GABA_AR$ is maintained. This astrocytic GABA-mediated $Cl^-$ efflux might help maintain the postsynaptic transmembrane $Cl^-$ gradients of GABAergic synapses by spatially buffering $[Cl^-]_o$.
